# Tepilamide Fumarate as a Novel Potentiator of Virus-Based Therapy

**DOI:** 10.3390/v16060920

**Published:** 2024-06-05

**Authors:** Akram Alwithenani, Rozanne Arulanandam, Boaz Wong, Marcus M. Spinelli, Andrew Chen, Glib Maznyi, Victoria H. Gilchrist, Tommy Alain, Jean-Simon Diallo

**Affiliations:** 1 Centre for Cancer Therapeutics, Ottawa Hospital Research Institute, Ottawa, ON K1H 8L6, Canada; 2 Department of Biochemistry, Microbiology, and Immunology, Faculty of Medicine, University of Ottawa, Ottawa, ON K1H 8M5, Canada; 3 Department of Clinical Laboratory Science, Faculty of Applied Medical Science, Umm Al-Qura University, Makkah 24382, Saudi Arabia; 4 Children’s Hospital of Eastern Ontario Research Institute, Apoptosis Research Center, Ottawa, ON K1H 8L1, Canada

**Keywords:** cancer therapeutics, tepilamide fumarate, VSVΔ51, viral vectors, gene therapy

## Abstract

Oncolytic virotherapy, using viruses such as vesicular stomatitis virus (VSVΔ51) and Herpes Simplex Virus-1 (HSV-1) to selectively attack cancer cells, faces challenges such as cellular resistance mediated by the interferon (IFN) response. Dimethyl fumarate (DMF) is used in the treatment of multiple sclerosis and psoriasis and is recognized for its anti-cancer properties and has been shown to enhance both VSVΔ51 and HSV-1 oncolytic activity. Tepilamide fumarate (TPF) is a DMF analog currently undergoing clinical trials for the treatment of moderate-to-severe plaque psoriasis. The aim of this study was to evaluate the potential of TPF in enhancing the effectiveness of oncolytic viruses. In vitro, TPF treatment rendered 786-0 carcinoma cells more susceptible to VSVΔ51 infection, leading to increased viral replication. It outperformed DMF in both increasing viral infection and increasing the killing of these resistant cancer cells and other cancer cell lines tested. Ex vivo studies demonstrated TPF’s selective boosting of oncolytic virus infection in cancer cells without affecting healthy tissues. Effectiveness was notably high in pancreatic and ovarian tumor samples. Our study further indicates that TPF can downregulate the IFN pathway through a similar mechanism to DMF, making resistant cancer cells more vulnerable to viral infection. Furthermore, TPF’s impact on gene therapy was assessed, revealing its ability to enhance the transduction efficiency of vectors such as lentivirus, adenovirus type 5, and adeno-associated virus type 2 across various cell lines. This data underscore TPF’s potential role in not only oncolytic virotherapy but also in the broader application of gene therapy. Collectively, these findings position TPF as a promising agent in oncolytic virotherapy, warranting further exploration of its therapeutic potential.

## 1. Introduction

Oncolytic viruses (OVs) are specialized therapeutic agents designed to selectively infect and destroy cancer cells, leaving healthy tissues unharmed [1,2]. These agents, through their multifaceted therapeutic mechanisms, not only directly induce the lysis of malignant cells but also potentiate antitumor immune responses and target the tumor vasculature [3], thereby comprehensively impeding tumor progression. A notable example is Talimogene laherparepvec (T-VEC), a Herpes Simplex Virus (HSV-1)-based oncolytic virus, which garnered FDA approval in 2015 for treating melanoma [4,5]. Other HSV-1-based oncolytic viruses like G47Δ are showing promise in clinical trials. In glioblastoma patients, G47Δ demonstrated safety and a notable 1-year survival rate of 84.2% in a phase 2 trial [6]. These results have led to its conditional and time-limited approval in Japan for glioma treatment, pending further studies.

Oncolytic virotherapy, while promising, encounters significant hurdles, particularly the resistance of cancer cells to viral infection, among other challenges. This resistance is frequently attributed to the host immune response and in part type I interferon (IFN) signaling [7]. Typically, cancer cells show a lack of interferon (IFN) reactivity as part of their neoplastic evolution; however, IFN signaling signatures can be observed in some tumor types and may be linked to OV resistance [8,9]. The IFN pathway which is induced among others by viral infection can activate a series of antiviral genes that effectively suppress viral replication. This innate immune mechanism, designed to protect cells from viruses, poses a challenge in oncolytic virotherapy, as it can significantly reduce the ability of therapeutic viruses to infect and destroy cancer cells. Overcoming this resistance is one key focus in the development of more effective oncolytic viral therapies.

To tackle the challenge of cancer cell resistance in oncolytic virotherapy, our team and others have tested the strategic use of selected pharmacological agents in conjunction with OVs. These strategies mostly aim to temporarily dampen the cancer cells’ IFN-related antiviral defenses, enhancing the effectiveness of oncolytic virotherapy. Multiple small molecules have been identified through high-throughput screening and rational approaches [10,11,12,13,14]. Many of these molecules target the IFN pathway and its downstream regulated genes, leading to increased viral titers and enhanced effectiveness in killing tumor cells. One notable example is Dimethyl fumarate (DMF), an FDA-approved drug for multiple sclerosis, which has shown significant potential in enhancing the activity of vesicular stomatitis virus (VSVΔ51) and various oncolytic HSV-1 strains in different cancer cell lines and mouse models [15]. Its effectiveness largely stems from its ability to inhibit the IFN type 1 pathway through preventing the nuclear translocation of NF-κB [14,16].

While DMF is an approved drug, it suffers from some known pharmacological limitations and side effects, which have led to the discovery and development of analogs with potentially more favorable pharmacokinetic and toxicological profiles. In this study, we focus on Tepilamide fumarate (TPF), one such analog that is currently under investigation in late-stage clinical trials for patients with psoriasis [17]. The unexplored potential of TPF in cancer treatment and in combination with OVs presents a significant opportunity to develop improved therapeutic regimens. Our study aimed to assess the anti-cancer potential of TPF, exploring its ability to enhance the effectiveness of oncolytic viruses and other viral vectors.

## 2. Materials and Methods

### 2.1. Drugs 

Dimethyl fumarate (DMF) and Monomethyl fumarate (MMF) were obtained from Sigma-Aldrich (St. Louis, MO, USA) (DMF: Cat#242926; MMF: Cat#651419). Tepilamide fumarate (TPF) was obtained from Dr. Reddy’s Laboratories Ltd. (Hyderabad, India). All the drugs were resuspended in 100% DMSO to 100 mM and further diluted at indicated dilutions before use in all in vitro experiments. 

### 2.2. Viruses 

VSVΔ51 expressing GFP or firefly luciferase (FLuc) was used throughout this study [18]. The propagation of all viruses was carried out on Vero cells and subsequently purified using 5–50% OptiPrep (Sigma-Aldrich, St. Louis, MO, USA) gradients. Viral titers were determined using a standard plaque assay on the Vero cells, following a published protocol [19].

HSV.n212 was obtained as a generous gift from Dr. Karen Mossman of McMaster University (Hamilton, ON, Canada). HSV.n212 titers were determined by a standard plaque assay on the Vero cells according to a previously published protocol [20].

AAV2 expressing GFP was obtained from Virica Biotech (Ottawa, ON, Canada).

Lentivirus plasmids were purchased from Thermo Fisher Scientific (Nepean, ON, Canada) (pLenti6.3 GFP expression vector, Cat# V37006, LV MAX Packaging Mix Cat# A43237) and used to transfect HEK293 Viral Production cells (Gibco, Nepean, ON, Canada, Cat#A35347) using the LV MAX kit (Cat# A35348) and quantified by flow cytometry.

Ad5 expressing GFP was purchased from Vector Biolabs (Malvern, PA, USA), amplified on HEK293 cells, purified by CsCl gradient, and quantified using the Adeno-X Rapid Titer Kit from Takara (Palo Alto, CA, USA).

### 2.3. Cell Lines 

All the cell lines used in this study along with information about their suppliers and the growth media are detailed in (Table S1). The cells were cultured in Dulbecco’s Modified Eagle’s Medium (DMEM), sourced from either HyClone (Waltham, MA, USA) or Corning (Manassas, VA, USA). This medium was supplemented with 1% penicillin–streptomycin (Gibco) and 10% fetal bovine serum (FBS), obtained from VWR (Mississauga, ON, Canada), to support optimal cell growth and maintenance.

All the cells were incubated at 37 °C in a 5% CO_2_ humidified incubator and routinely tested for mycoplasma contamination by Hoechst staining and PCR (Diamed, Mississauga, ON, Canada, Cat # ABMG238).

### 2.4. Plaque Assay

The Vero cells were initially cultured in 12-well plates at a specific density of 3 × 10^5^ cells per well. The infectious samples underwent a series of dilutions using serum-free DMEM and were subsequently applied (at a volume of 500 µL per well) to the Vero cell monolayers. These cultures were then incubated at 37 °C, 5% CO_2_ for a duration of 60 min. Following this incubation period, the culture medium was removed and replaced with an overlay. This overlay was prepared by mixing 1% agarose and 2× DMEM supplemented with 20% FBS in a 1:1 ratio. After an additional 24 h incubation, visible plaques were fixed with a mixture of methanol and glacial acetic acid in a 3:1 ratio for a minimum of 1 h, and then stained for 30 min with a Coomassie Blue solution (comprising 4 g of Coomassie Brilliant Blue R (Sigma, Oakville, ON, Canada) (cat. B0149), 800 mL of methanol, 400 mL of acetic acid, and 2800 mL of distilled water) to enable the visualization and counting of plaques.

### 2.5. High-Throughput Luciferase Tittering

The Vero cells were cultured in opaque white 96-well plates using 100 μL of complete DMEM supplemented with 30 mM HEPES until they reached a confluence of 95–100%. To determine the viral titer of VSVΔ51-Fluc-infected samples, 25 μL of each sample was transferred into the wells containing the Vero cells. Additionally, a standard curve was prepared by diluting a purified virus stock with a known titer ranging from 10^6^ to 10^9^ PFU/mL. This standard curve was duplicated for each 96-well plate. D’Luciferin (PerkinElmer, Waltham, MA, USA, Cat. # 122799) solution was added to the wells (2 mg/mL) in sterile PBS. Luminescence was read using a Cytation microplate reader at an appropriate sensitivity. Standard curve values allow for the generation of a Hill equation, which was applied to the tittered samples to obtain viral expression units (VEU). Further details can be found in our published protocol [21].

### 2.6. Viral Growth Curves

The cells were placed in 24-well plates and allowed to grow overnight, reaching full confluency by the following day. The cells were then inoculated with VSVΔ51-Fluc at an MOI of 0.01 (multi-step growth curve) or 1.0 (single-step growth curve). The cells were incubated up to 48 hpi, with 200 µL of supernatant collected and frozen at −80 °C at the following timepoints: 0, 8, 12, 24, 36, and 48 hpi. Viral titers in the collected samples were quantified by plaque assay as previously described.

### 2.7. Cell Viability Assay

Cellular metabolic activity was evaluated using the Resazurin metabolic dye (Millipore Sigma, Oakville, ON, Canada, cat. SI03200) following the manufacturer’s instructions. Resazurin solution with a concentration of 10% (*v*/*v*, final) was added to the wells containing treated or untreated and/or infected cells. The cells were then incubated for a duration of 2–4 h, which varied depending on the specific cell line used. Using the BioTek Microplate Reader (BioTek, Winooski, VT, USA) and Gen5 2.07 software, fluorescence was measured at 590 nm upon excitation at 530 nm. The readings were expressed relative to the average of the uninfected, untreated condition.

### 2.8. IFN-β ELISA

The 786-0 cells were plated to confluency in 12-well plates and incubated overnight at 37 °C in a 5% CO_2_ humidified incubator. Subsequently, the cells were treated with TPF at a concentration of 150 µM. Four hours later, the cells were either infected with VSVΔ51 at a multiplicity of infection (MOI) of 0.05 or underwent a mock infection. Supernatants were collected at 24 h post-infection (hpi). The quantity of human IFN-β in the supernatant was measured using the Human IFN Beta ELISA Kit (PBL Assay Science, Piscataway, NJ, USA, cat. 41410), following the procedure provided by the manufacturer. Absorbance readings were taken using the BioTek Microplate Reader (Agilent, Santa Clara, CA, USA).

### 2.9. Human and Murine Ex Vivo Models

BALB/c mice were implanted with CT26.wt 3 × 10^5^ colon cancer cells. Once tumor volumes reached 1500 mm^3^, the mice were euthanized, and relevant tissues were extracted. For human tissue samples, tumor samples were collected from patients who had given informed consent and followed the Declaration of Helsinki guidelines during surgical resection. The collection of human tissue/fluid for this study was made possible by the Global Tissue Consent and Collection Program at the Ottawa Hospital Research Institute. All the tissues were sliced into 2 mm sections and circular cores of 2 mm diameter were extracted using a punch biopsy tool. These cores were then kept in a humidified incubator at 37 °C and 5% CO_2_ in DMEM supplemented with 10% serum, 30 mM HEPES, 1% (*v*/*v*) penicillin–streptomycin and 0.25 mg/L amphotericin B. The cores were treated with TPF for four hours and then infected with VSVΔ51 at 3 × 10^4^ pfu/core. After 24 h post-infection, fluorescence images were captured using the EVOS Live Cell Imaging System (Thermo Fisher, San Francisco, CA, USA) the supernatant was assessed for viral titer by standard plaque assay.

### 2.10. Quantitative Real-Time PCR

The extraction of total RNA from both infected and mock-infected 786-0 cells was conducted using the RNeasy Mini Kit, following the instructions provided by the manufacturer. (Qiagen, Toronto, ON, Canada, Cat. 74104). The conversion of one microgram of RNA to complementary DNA (cDNA) was achieved through the use of the RevertAid First Strand cDNA Synthesis Kit. (Thermo Fisher Scientific, Nepean, ON, Canada, Cat. K1621). The real-time polymerase chain reaction (PCR) protocols involved the use of 40 nanograms of complementary DNA (cDNA) and the PowerUp™ SYBR™ Green Master Mix (ThermoFisher Scientific, Cat. A25776) on the 7500 Fast Real-Time PCR system (Applied Biosystems, Thermo Fisher Scientific, Nepean, ON, Canada). Gene expression was normalized to GAPDH, and fold change was calculated relative to the mock-treated samples for each gene using the Pfaffl method [22].

### 2.11. Immunofluorescence Staining

The 786-0 cells were seeded on 12 mm glass coverslips (Thomas Scientific, cat. 64-0712), then treated with TNFa (Sigma-Aldrich, Oakville, ON, Canada) or TPF. Next, the adherent cells were washed twice using PBS* containing 1 mM CaCl_2_ and 500 mM MgCl. The cells were subjected to fixation using a 4% paraformaldehyde solution for a duration of 30 min. Subsequently, permeabilization was achieved by treating the cells with a 0.2% Triton-X 100 solution in 200 mM glycine/PBS for a period of 8 min. The cells were quenched in a 200 mM glycine/PBS solution. Following this, the samples underwent blocking with a 5% BSA/PBS* solution for 1 h at room temperature and were subsequently exposed to the NF-κB/p65 primary antibody (rabbit, cell signaling #8242) overnight in a humidified chamber at 4 °C. Anti-rabbit IgG secondary antibody (cell signaling #4413S) was applied for 60 min, then the samples were mounted onto glass slides and counterstained using Prolong gold antifade with DAPI (Molecular Probes). The slides were imaged using the Zeiss Axiocam HRM Inverted fluorescent microscope (Zeiss, Toronto, Canada) and Axiovision 4.0 software. The images were processed using ImageJ. Nuclear: cytoplasmic signal quantification processes were performed using CellProfiler (Massachusetts Institute of Technology, Cambridge, MA, USA).

### 2.12. Viral Vector Transduction

Dose–response testing of TPF or DMF followed by transduction with either Lentivirus, AAV-2, or Ad5 was performed in triplicate in the indicated cell lines seeded in 96-well plates to 50% confluency. The cells were treated with a 2 in 3 dilution series of each drug for 4 h followed by transduction with either lentivirus-GFP at MOI 10, AAV2-GFP at MOI 5000, or Ad5-GFP at MOI 5–100. A total of 70 h post transduction, the plates were imaged for GFP using the Cellomics Arrayscan (Thermo Scientific, Waltham, MA, USA), and mean fluorescence per well was determined using the Agilent Biotek Cytation 5 reader (Santa Clara, CA, USA).

### 2.13. Statistics

All the graphs and statistical analyses in our study utilized GraphPad Prism v.10. The specific statistical tests employed for each figure are described in the corresponding figure legends. When comparing the means of two groups, a two-tailed unpaired Student’s *t*-test was applied. For comparisons involving more than two groups, one-way ANOVA with either Dunnett’s or Tukey’s multiple correction tests was used. The number of biological replicates is denoted by ‘n’, with the error represented as the standard error of the mean (SD). A *p*-value less than 0.05 was considered to indicate statistical significance.

## 3. Results

### 3.1. TPF Enhances Susceptibility of Cancer Cells to VSVΔ51 Infection

**Figure 1:**
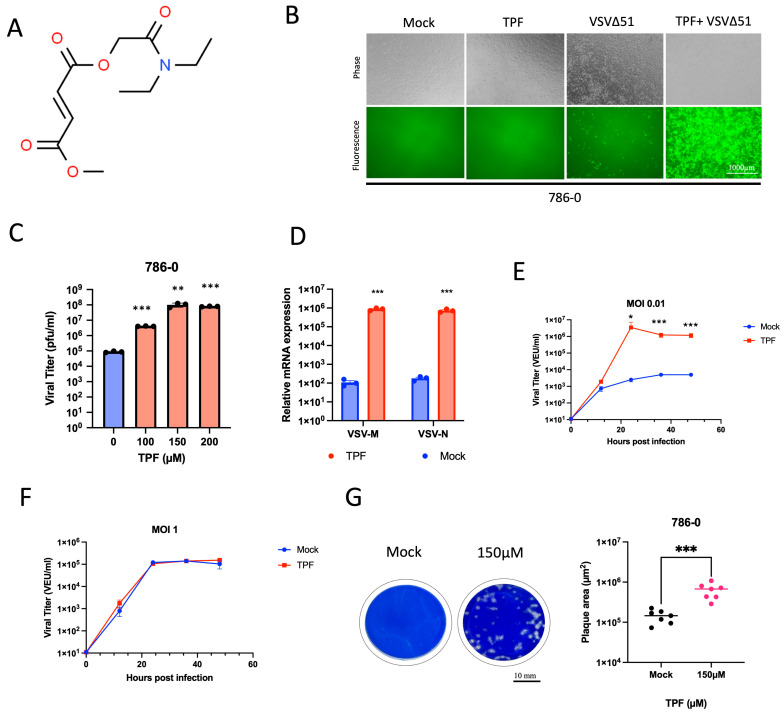
Tepilamide fumarate demonstrates remarkable viral sensitizing capabilities. (**A**) The structure of Tepilamide fumarate (TPF). The 786-0 cells were pre-treated with different doses (0, 100, 150, and 200 µM) of TPF for four hours and then infected with VSVΔ51 expressing GFP (MOI 0.05). (**B**) GFP images were captured 24 h post-infection. (**C**) The samples were quantified for viral titer by standard plaque assay (N = 3, mean ± SD; ** *p* < 0.01 *** *p* < 0.001; one-way ANOVA with Dunnett’s multiple comparisons test compared to Mock). (**D**) VSV-M and VSV-N mRNA were quantified by RT-qPCR (N = 3, mean ± SD; *** *p* < 0.001 by Student’s *t*-test). Multistep (MOI 0.01) and single-step (MOI 1) growth curves were generated. (**E**,**F**) The 786-0 cells were pre-treated with TPF and infected with VSVΔ51 at an MOI of 0.01 or 1, and the samples were quantified for viral titer by high throughput method (N = 3; mean ± SD; * *p* < 0.05, *** *p* < 0.001; two-tailed *t*-test). (**G**) The 786-0 cells underwent treatment with TPF at a concentration of 150 µM, followed by infection with VSVΔ51 at an MOI of 0.01. After an incubation period of 1 h, the cells were overlaid with agarose. At 48 h post-infection, Coomassie blue staining was performed. The area of randomly selected plaques was then measured (N = 7), and a two-tailed *t*-test revealed a highly significant difference (*** *p* < 0.001).

To delineate the virus-enhancing potential of TPF (Figure 1A), we utilized human renal 786-0 carcinoma cells that are inherently resistant to VSVΔ51 infection as a model system. The 786-0 cells were subjected to pre-treatment with TPF at different concentrations for a duration of four hours. Subsequently, these cells were infected with VSVΔ51 that expresses GFP. Fluorescence microscopy pictures captured 24 h after infection, as depicted in (Figure 1B), revealed that TPF pre-treatment increases VSVΔ51 infection in these cells compared to untreated, infected controls. Similar outcomes were noted when 786-0 cells were pre-treated with other drugs from the fumarate class, including DMF and the active metabolite of TPF and DMF, Monomethyl fumarate (MMF), as demonstrated in (Figure S1). A significant increase in viral titer with rising concentrations of TPF in the 786-0 cells was observed (Figure 1C), suggesting a dose-dependent enhancement of viral growth upon treatment. Additionally, TPF was used to pre-treat other cancer cell lines, such as MC38, 4T1, and B16-OVA, and we observed a similar increase in GFP transgene expression following VSVΔ51 infection, as demonstrated in (Figure S2). Quantitative PCR analysis confirmed that the expression levels of the VSV M and N genes were significantly increased in the RNA samples. This supports the presence of greater viral loads in cells exposed to TPF compared to the cells that were not treated as shown in (Figure 1D). In addition, we assessed the enhancing impact of TPF on VSVΔ51 infectivity by comparing multi-step and single-step growth curves. Consistent with our previous findings with DMF [15], TPF significantly increased the infectivity of VSVΔ51 at a low MOI of 0.01, compared to a high MOI of 1. This was determined using a high-throughput titration titer quantification method, as shown in (Figure 1E,F). These findings suggest that TPF enhances viral spread, contributing to augmented growth without necessarily affecting the VSVΔ51 replication rate. The spread of the virus was also evaluated using a plaque assay designed to measure plaque size, revealing that TPF significantly increased the area of viral plaques within a monolayer of the 786-0 cells, as determined by Coomassie blue staining as shown in (Figure 1G).

### 3.2. TPF Enhances OV Infection in Cancer Cells Compared to DMF

**Figure 2:**
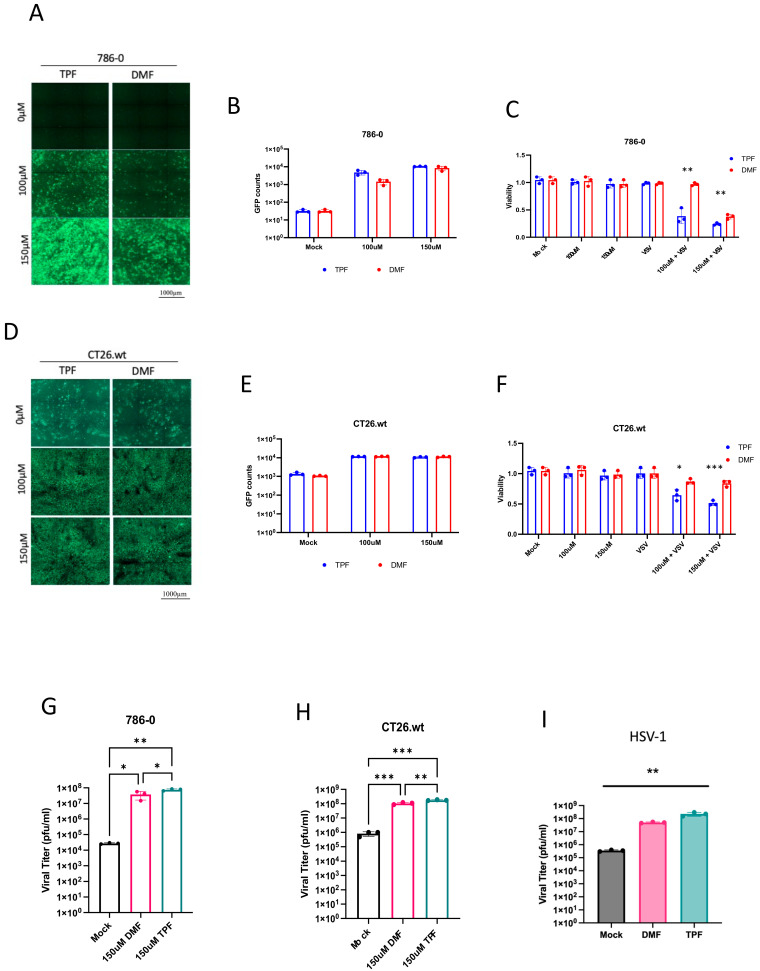
TPF demonstrates a superior effect in enhancing OV infection within cancer cells. (**A**,**B**,**D**,**E**) 786-0 and CT26.wt cells were first pre-treated with TPF and DMF (100 µM and 150 µM) for a duration of 4 h then infected with VSVΔ51 at MOI (0.05). The 24 h post infection GFP counts were counted (N = 3, mean ± SD). (**C**,**F**) Metabolic viability assay was assessed using Resazurin dye (N = 3, mean ± SD; * *p* < 0.05, ** *p* < 0.01, *** *p* < 0.001; unpaired *t*-test). (**G**,**H**) The samples were quantified for viral titer by standard plaque assay (N = 3, mean ± SD; * *p* < 0.05, ** *p* < 0.01 *** *p* < 0.001; one-way ANOVA with Tukey’s multiple comparisons test). (**I**) The 786-0 cells were treated with either TPF or DMF concentration of 150 µM then infected with HSV.n212 expressing GFP (MOI 0.02). Corresponding fluorescent images were taken 48 h post infection. Supernatants were collected at 72 h post infection for viral titer quantification by plaque assay (mean ± SD; ** *p* < 0.01 by one-way ANOVA).

Given the enhancement of oncolytic activity observed with TPF in conjunction with VSVΔ51 and considering previous studies demonstrating DMF’s ability to potentiate VSVΔ51 efficacy [15], our study aimed to conduct a direct comparison between DMF and TPF to determine if one drug exhibits superior effectiveness in vitro. 786-0 cells were pre-treated with TPF or DMF for four hours prior to infection with VSVΔ51-GFP at MOI of 0.05. Subsequent to a 24 h infection period, fluorescence imaging was performed, and GFP expression was quantified as illustrated in (Figure 2A). The acquired images and corresponding GFP quantification indicate that both TPF and DMF, at 100 and 150 μM, significantly increase VSVΔ51-GFP associated GFP expression, suggesting an augmentation in viral infectivity in response to compound administration.

Further analysis aimed to evaluate the impact of TPF and DMF on VSVΔ51-mediated oncolysis. Cell viability was measured using the metabolic indicator alamarBlue at 48 h post-treatment, with and without the addition of VSVΔ51 to the TPF or DMF treatment regimens. The results, as depicted in (Figure 2C), demonstrate a marked decline in cell viability following the combination therapy, underscoring the compounds’ potential to compromise the viability of the VSV-resistant 786-0 cancer cell line. A side-by-side comparison of the TPF-VSVΔ51 and DMF-VSVΔ51 combinations, detailed in (Figure 2C), reveals a superior reduction in cell viability by the TPF-VSVΔ51 regimen, particularly at 100µM, suggesting that TPF may exert a more pronounced and potent oncolytic effect in the presence of VSVΔ51 than DMF.

This pattern of reduced cell viability by the TPF-VSVΔ51 combination over the DMF- VSVΔ51 combination was also observed in another cell line, the CT26.wt mouse colon carcinoma cell line, as shown in (Figure 2D–F). These results further support that the TPF-VSVΔ51 combination further improves cancer cell elimination in comparison to the DMF- VSVΔ51 combination.

Resulting viral titers were also quantified, as shown in (Figure 2G,H), and revealed a significant increase in viral titers in both 786-0 and CT26.wt cells treated with either the TPF or DMF combination treatments. A slightly greater but statistically significant enhancement in impact on virus titers was observed with TPF-VSVΔ51 compared to DMF- VSVΔ51. To evaluate the effects of TPF in the context of another OV class, HSV.n212 was tested in combination with either TPF or DMF in the 786-0 cells. The results indicate once again a slight superiority of TPF over DMF in enhancing the growth of HSV.n212, as shown in (Figure 2I). The data across these figures demonstrate that TPF, especially in combination with VSVΔ51, has a potent ability to augment cancer cell infection and killing across diverse cellular models.

### 3.3. TPF Potentiates Infectivity of VSVΔ51 in Human and Murine Tumor Explants

**Figure 3:**
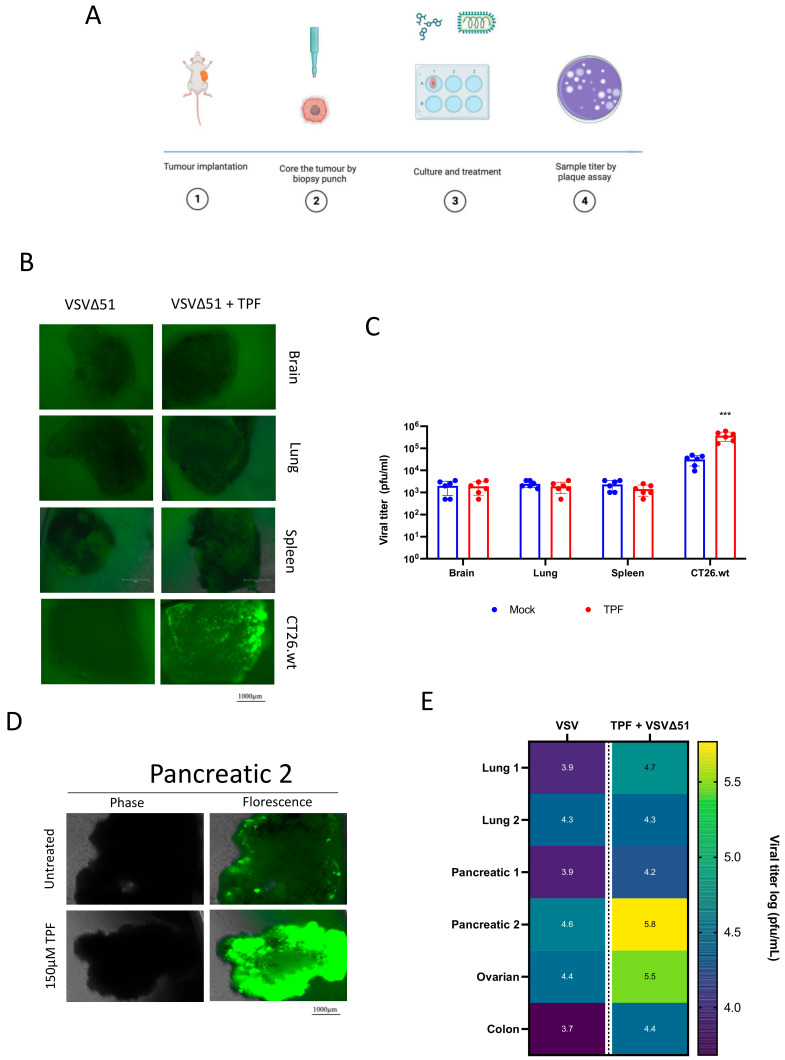
Tepilamide fumarate promotes VSVΔ51 infection in ex vivo models. (**A**) The schematic diagram illustrates the methodology of the coring experiment. CT26.wt colon tumors were grown in BALB/c mice, and once reaching a volume of 1500 mm^3^m the tissues were collected and cored. Clinical specimens were obtained and cored. The cores were treated with TPF and then infected with VSVΔ51 3 × 10^4^ (pfu/core). (**B**,**D**) Fluorescent images were taken 24 h post infection, and (**C**,**E**) supernatants were collected at 24 hpi for viral titer quantification by plaque assay (n > 5, mean ± SD; *** *p* < 0.001 by two-tailed *t*-test).

We next aimed to evaluate the impact of TPF in ex vivo tissues to assess its potential in a more physiologically relevant setting and to assess its tumor selectivity, a feature that has been previously established for DMF [15]. Briefly, CT26.wt murine colon cancer cells were implanted subcutaneously in mice, and once the tumors had grown to approximately 1500 mm^3^ these were harvested and cored for in vitro culture using uniform slices from a biopsy punch (Figure 3A) [23]. Samples from normal tissues including brain, spleen, and lung, were also similarly harvested. All the cancer and normal tissue samples in culture were then exposed to VSVΔ51, with and without the pre-addition of TPF at a concentration of 150 µM. The resulting fluorescence images and viral titer data in (Figure 3B,C) reveal a marked increase in VSVΔ51 infectivity in CT26.wt tumor cores following TPF pre-treatment. Conversely, normal brain and other tissues did not exhibit increased VSVΔ51 levels, demonstrating that the virus’s tumor-specific selectivity is preserved despite TPF pre-treatment.

Analogous human cancer explant experiments were subsequently performed; wherein consenting patient tumor specimens were obtained from the clinic freshly and were similarly processed. A regimen of TPF pre-treatment followed by VSVΔ51 infection was administered across multiple tumor types, including pancreatic, lung, ovarian, and colon tumors. The results detailed in (Figure 3D,E); Table S1 indicate a marked variation in TPF’s efficacy to potentiate viral infectivity. Notably, one pancreatic tumor explant demonstrated the most substantial response with a fifteen-fold increase in VSVΔ51 output post-TPF pre-treatment. An ovarian tumor also responded substantially, showing a ten-fold enhancement in viral replication. These findings collectively indicate that TPF selectively enhances the infectivity of VSVΔ51 but with some variation across human and murine tumor explants.

### 3.4. TPF Inhibits the Type-I IFN Response and NF-κB to Amplify VSVΔ51 Oncolytic Potency

Given the established capacity of DMF to downregulate the interferon (IFN) pathway and interferon-stimulated genes [15], we endeavored to investigate whether TPF exerts comparable modulatory effects on these critical components of the antiviral response.

**Figure 4:**
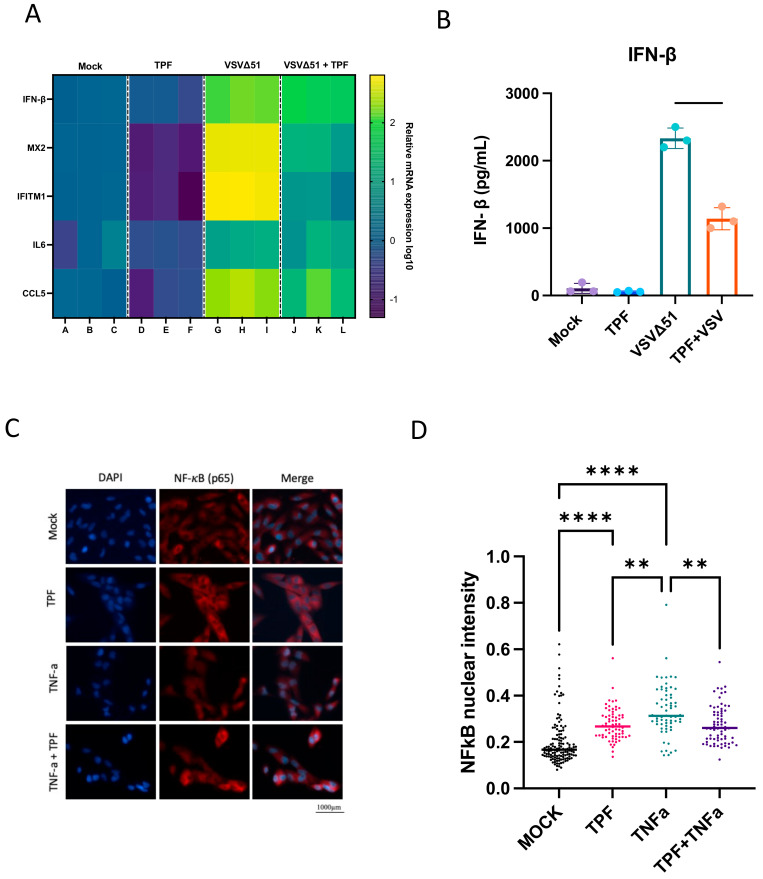
Tepilamide fumarate promotes VSVΔ51 infection via type 1 IFN inhibition. (**A**) The 786-0 cells were pre-treated for 4 h with TPF (150 μM) then infected with VSV∆51 (MOI 0.05), and 24 h post infection RNA was extracted and mRNA for indicated genes was quantified by RT-qPCR. (**B**) Supernatants were taken 24 hpi and assayed for IFN-β by ELISA. (N = 3, mean ± SD; **** *p* < 0.0001; one-way ANOVA with Tukey’s multiple comparisons test). The 786-0 cells were pre-treated for 4 h with TPF (150 μM) and then infected with VSV∆51 (MOI 1) for 6 h. The cells were fixed and immunostained for NF-κB and nuclei (DAPI). (**C**) Representative fluorescent images were taken. (**D**) Nuclear NF-κB intensity was quantified. (N = 3; mean ± SD; ** *p* < 0.01, **** *p* < 0.0001 one-way ANOVA with Tukey’s multiple comparisons test).

Utilizing quantitative PCR (qPCR), we measured the transcription levels of key antiviral and pro-inflammatory genes—specifically IFN-β, MX2, IFITM1, IL6, and CCL5—in the 786-0 cells subjected to TPF (150 µM) treatment or vehicle. Following a four-hour incubation, these cells were either infected with VSVΔ51 or left uninfected. Our findings demonstrate that the TPF treatment leads to a significant downregulation of the above antiviral and pro-inflammatory genes as shown in (Figure 4A). In addition to qPCR analyses, an enzyme-linked immunosorbent assay (ELISA) was conducted to quantify IFN-β in the supernatant of cell cultures. The results corroborated the qPCR findings, with TPF demonstrating a consistent downregulation of IFN-β secretion as shown in (Figure 4B). This further confirms that TPF modulates the antiviral response in 786-0 cells, enhancing their susceptibility to VSVΔ51 infection and underscoring the potential of TPF as a facilitator in oncolytic virus therapy. In line with the suppression of the type I interferon response, TPF treatment led to a reduction in the activation (indicated by phosphorylation) of both STAT1 (signal transducer and activator of transcription 1) and STAT2, observed 24 h post-infection (Figure S3).

Building on previous studies demonstrating that DMF inhibits the nuclear translocation of the p65 subunit of NF-κB [15], we aimed to evaluate whether TPF exhibits a similar inhibitory effect. Indeed, immunofluorescence staining (Figure 4C,D) shows a suppression of TNF-α-stimulated NF-κB nuclear translocation (p65) in the cells pre-treated with TPF, suggesting that TPF, like DMF, may impede NF-κB signaling in the context of inflammatory stimulation. However, we did note that TPF on its own did lead to elevated NF- κB nuclear translocation, something that has not been previously observed with DMF [15].

### 3.5. TPF Amplifies Transduction Efficiency across Multiple Viral Vectors

Following the observed enhancement of oncolytic virus activity by TPF, we sought to examine the influence of TPF on other viral vectors, particularly non-replicating vectors used in gene therapy. This included lentivirus, adenovirus type 5 (Ad5), and adeno-associated virus type 2 (AAV2). The aim was to unravel TPF’s potential in boosting the transduction efficiency of these vectors, potentially expanding the utility of TPF for other gene therapy techniques.

**Figure 5:**
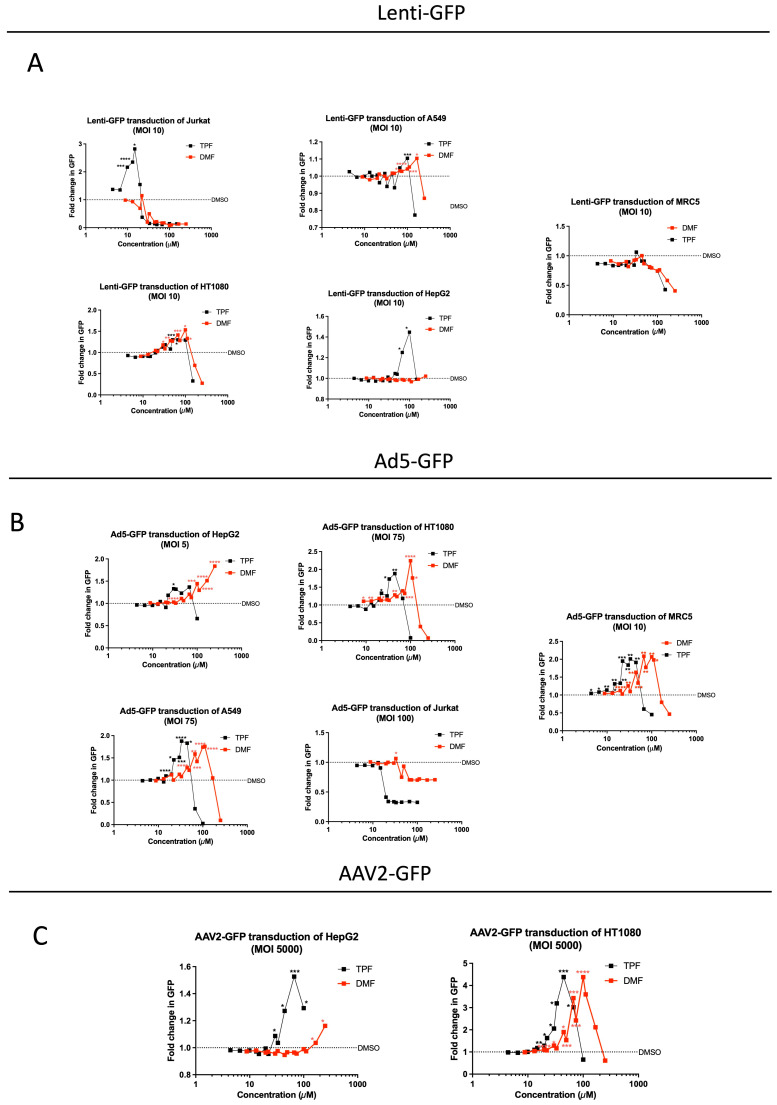
The impact of TPF in the transduction of lent-GFP, AVV-2, and Ad5-GFP. (**A**) Jurkat, HT1080, A549, HepG2, or MRC5 cells were seeded in 96-well plates and pre-treated for 4 h with 4–100 μM TPF (black lines) or 9–250 μM DMF (red lines) in triplicate. The cells were transduced with lentivirus encoding GFP at an MOI of 10. A total of 70 h post transduction, the plates were imaged for GFP and mean fluorescence per well determined using the Agilent Biotek Cytation5. The GFP mean intensity was quantified and plotted (N = 3, unpaired *t*-test was performed comparing to the DMSO-treated, transduced condition). (**B**) Jurkat, HT1080, A549, HepG2, or MRC5 cells were seeded in 96-well plates and pre-treated for 4 h with 4–100 μM TPF (black lines) or 9–250 μM DMF (red lines) in triplicate. The cells were transduced with Ad5 encoding GFP at the indicated MOIs. A total of 70 h post transduction, the plates were imaged for GFP and mean fluorescence per well determined using the Agilent Biotek Cytation5. GFP mean intensity was quantified and plotted (N = 3, unpaired *t*-test was performed comparing to the DMSO-treated, transduced condition). (**C**) HT1080 or HepG2 cells were seeded in 96-well plates and pre-treated for 4 h with 4–100 μM TPF (black lines) or 9–250 μM DMF (red lines) in triplicate. The cells were transduced with AAV2 encoding GFP at the indicated MOIs. A total of 70 h post transduction, the plates were imaged for GFP and mean fluorescence per well determined using the Agilent Biotek Cytation5. GFP mean intensity was quantified and plotted (N = 3, unpaired *t*-test was performed comparing to the DMSO-treated, transduced condition. * *p* < 0.05, ** *p* < 0.01, *** *p* < 0.001, **** *p* < 0.0001).

We initially evaluated the ability of TPF and DMF to enhance the transduction efficiency of lentivirus across a spectrum of cell lines, including MRC5, HT1080, HepG2, A549, and Jurkat. Following a 4 h pre-treatment with a broad, non-toxic dose range of each compound and the transduction of cells with a GFP-expressing lentivirus at MOI 10, our observations revealed that TPF, alongside DMF, exhibited a notable increase in the transduction of lentivirus in several cell lines. At peak dosing (after which cell toxicity was observed), TPF significantly increased lenti-GFP-associated fluorescence in HepG2 (100 and 67 µM), HT1080 (67 and 44 µM), A549 (100 µM), Jurkat (10–20 µM), while DMF’s effect was only statistically significant in HT1080 (~50–120 µM), A549 (~100–150 µM) (Figure 5A). Remarkably, in Jurkat cells, TPF significantly boosted lentivirus transduction by ~3-fold at a peak concentration of 14 µM, as depicted in (Figure 5A), while DMF did not exhibit any notable effect under similar conditions.

We used the same cell lines to further assess the impact of TPF and DMF on the transduction efficiency of Ad5-GFP. Here, we observed that both TPF and DMF significantly increased Ad5-GFP transduction in several cell lines 70 h post transduction. TPF increased Ad5-GFP-associated fluorescence in MRC5 (peak ~ 30 µM), A549 (peak ~ 30 µM), HT1080 (peak ~ 50 µM), and HepG2 (peak ~ 50 µM). DMF also increased Ad5-GFP-associated fluorescence in MRC5 (peak ~ 100 µM), A549 (peak ~ 110 µM), HT1080 (peak ~ 100 µM), and HepG2 (peak > 250 µM). Altogether, we observed a significant difference in their peak effective concentrations, with TPF exhibiting better potency for this virus in all the cell lines where there was an increase in transduction observed as illustrated in (Figure 5B).

Lastly, we examined the effects of TPF and DMF on the transduction of AAV2-GFP in HT1080 and HepG2 cell lines. In both cell lines, but most prominently in HT1080 cells, TPF demonstrated a notable increase in AAV2-GFP transduction efficiency achieving >4-fold enhancement at peak concentration (between 22–44 µM, Figure 5C). In both cell lines, DMF potency was reduced compared to TPF. Altogether, our results suggest that TPF has the potential to be useful more broadly as a potentiator of viral vector-based therapeutics, and demonstrates greater in vitro potency than DMF in enhancing infection, a trend observed across a diverse array of cell lines.

## 4. Discussion

Our study shows for the first time that TPF has the potential as a novel pharmacological tool to enhance the activity of oncolytic viruses and other viral vectors. While the value of DMF has been previously explored to this end [15], the current study suggests that TPF has the potential for greater potency for this purpose as shown for a range of viruses in multiple cell lines (Figure 2 and Figure 4).

Mechanistically, our previous work with multiple maleic and fumaric acid esters, including MMF, the common active metabolite of TPF and DMF, has established the ability of these molecules to downregulate several IFN pathway-related genes via the blocking of NF-κB nuclear translocation [15]. Based on these findings, we hypothesized that TPF might target similar pathways. Our results provide clear evidence that TPF downregulates key antiviral and pro-inflammatory genes, including IFN-β, MX2, and IL6 (Figure 4A), thereby enhancing the susceptibility of cancer cells to VSVΔ51 infection. This downregulation, coupled with TPF’s inhibition of the NF-κB nuclear translocation induced by TNFα (Figure 4C), suggests that much like DMF, TPF modulates innate immune responses, creating a more favorable environment for viral infection and oncolysis in the context of replicating oncolytic viruses. Intriguingly, we also saw that TPF on its own led to an elevation of baseline NF-κB nuclear translocation (Figure 3D), which to date has not been observed with DMF [15].

While significant differences in oncolytic (i.e., cytotoxic) potency were observed between TPF and DMF, the difference in enhancement in total viral output comparing TPF and DMF was in contrast relatively limited. The reason this is intriguing is because this difference is seemingly insufficient to explain the comparatively large differences in oncolysis observed, even when looking at each drug’s respective peak viral enhancing dose. Given the dual role of NF-κB in both coordinating the antiviral response as well as cell survival and death, it is tempting to speculate that a shift in baseline NF-κB induced by TPF could contribute to the enhanced impact of TPF on oncolytic activity (Figure 4B,D).

While the antiviral response is a well-appreciated tumor resistance barrier in oncolytic virotherapy, our study suggests that this response also impedes the infection efficiency of other viral vectors, even those that do not effectively replicate. The extension of our study to evaluate TPF’s influence on different viral vectors provides support for its broader potential application in gene therapy. Engineered replication defective lentiviruses, adenoviruses, and adeno-associated viruses are prominent vectors being evaluated and used clinically in cell therapy (eg., Kymriyah™), gene therapy (e.g., Luxturna™), and even vector-vaccine applications (e.g., COVISHIELD™). Interestingly, each of these vectors uses distinct mechanisms for cellular attachment, entry, gene delivery, and in some cases integration (i.e., lentivirus). This is perhaps not surprising given that diverse viruses are commonly sensitive to antiviral responses mediated via type I IFN. However, given the viruses are non-replicating, further studies will be required in order to better understand what stage(s) of the infection process is / are most affected by baseline and/or emergent antiviral responses. Evidently, there are also cell-type and virus-type dependencies in relation to the effect of TPF which are not fully understood, since some cell lines were not sensitized to infection by select viruses using TPF (e.g., MRC5 for lentivirus and Jurkat for Ad5, Figure 5A and Figure 5B, respectively). Further, the effects of TPF on oncolytic virus replication and spread, analogously to DMF, were restricted to tumor cells.

Nevertheless, the potential of TPF as a potentiator of oncolytic virus and viral vector-based gene delivery more generally warrants further pre-clinical exploration. TPF-mediated enhancement could lead to more efficient gene transfer, potentially increasing the therapeutic efficacy of oncolytic virotherapies (as has been demonstrated with DMF previously) as well as gene therapies, supporting their clinical advancement. OVs as standalone treatments in particular have faced many challenges in delivering substantially improved clinical outcomes for cancer patients, with only a handful of approvals globally. Viral vector potentiators including but not limited to TPF could be considered in order to overcome some of these challenges. While our study included only a limited number of patient specimens, the observation of substantial activity of TPF with oncolytic VSVΔ51 in human pancreatic and ovarian cancer tumor explants (Figure 3E) offers choice areas for further pre-clinical exploration.

## Data Availability

All relevant data are provided within the paper and the Supplementary Files or available upon request.

## Supplementary Materials

The following supporting information can be downloaded at: https://www.mdpi.com/article/10.3390/v16060920/s1.
