# Supplementary material for: Tepilamide Fumarate as a Novel Potentiator of Virus-Based Therapy
Source: Viruses. 2024 Jun 5;16(6):920. doi: 10.3390/v16060920 (PMC11209164; doi:10.3390/v16060920)
Supplement: Supplementary file 1 [file viruses-16-00920-s001.zip › viruses-3029725-supplementary.pdf]

Supplementary Material

Tepilamide Fumarate as a Novel Potentiator of Virus-Based Therapy

Akram Alwithenani [1,2,3], Rozanne Arulanandam [1], Boaz Wong [1,2], Marcus M. Spinelli [1], Andrew Chen [1], Glib Maznyi [1], Victoria H. Gilchrist [1,2,4], Tommy Alain [2,4] and Jean-Simon Diallo [1,2,*]

[1] Centre for Cancer Therapeutics, Ottawa Hospital Research Institute, Ottawa, ON K1H 8L6, Canada
[2] Department of Biochemistry, Microbiology, and Immunology, Faculty of Medicine, University of Ottawa, Ottawa, ON K1H 8M5, Canada
[3] Department of Clinical Laboratory Science, Faculty of Applied Medical Science, Umm Al-Qura University, Makkah 24382, Saudi Arabia
[4] Children's Hospital of Eastern Ontario Research Institute, Apoptosis Research Center, Ottawa, ON K1H 8L1, Canada
* Correspondence: jsdiallo@ohri.ca

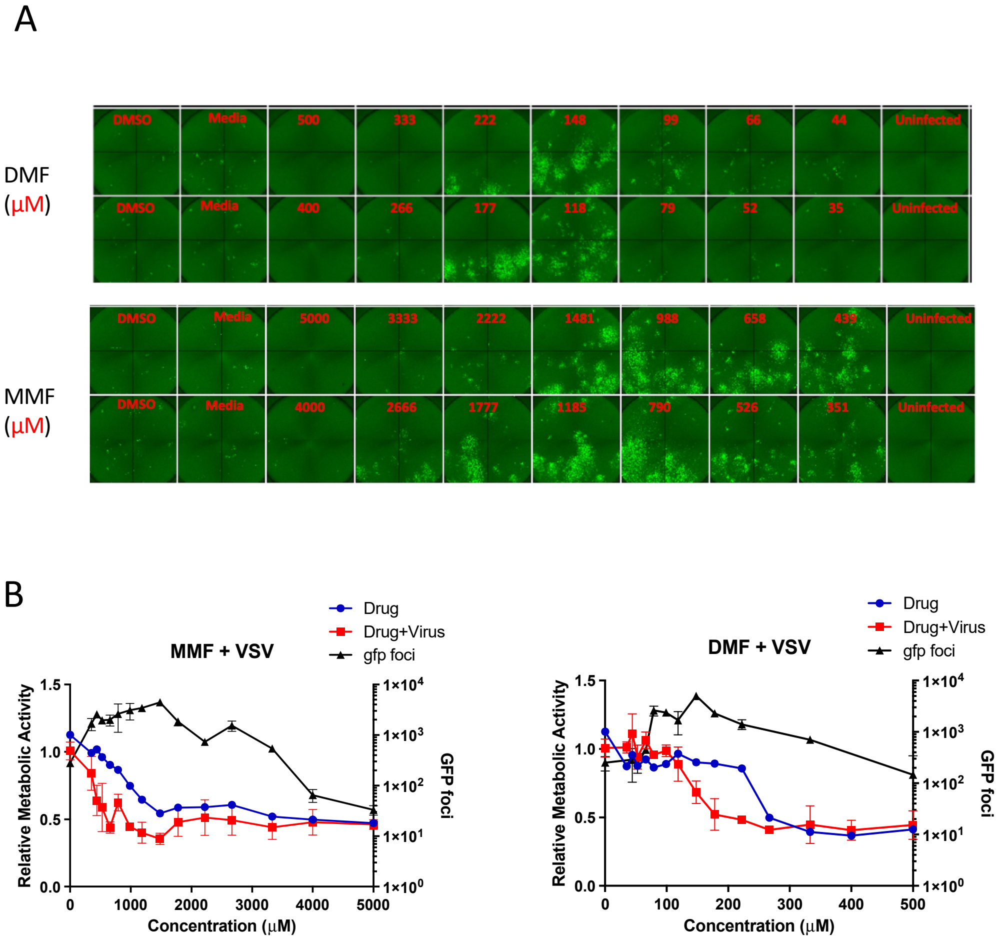

Figure S1: The impact of DMF, MMF on 786-0 cells.

786-0 cells were treated with indicated drugs with a range of concentrations then infected with VSVΔ51 expressing GFP (MOI 0.05). Corresponding fluorescent images were taken 24 hours post infection as shown in (A). GFP was quantified with a Cellomics ArrayScan® VTI HCS Reader (Thermo Fisher Scientific). Cell viability was assessed in all cells tested 48 hours after infection. The results were adjusted relative to the average values recorded for the respective uninfected and untreated cells as shown in (B).

A

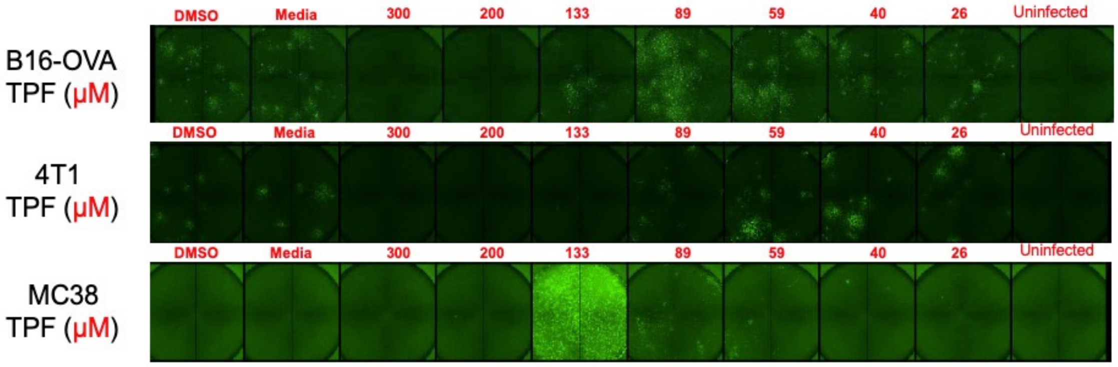

B

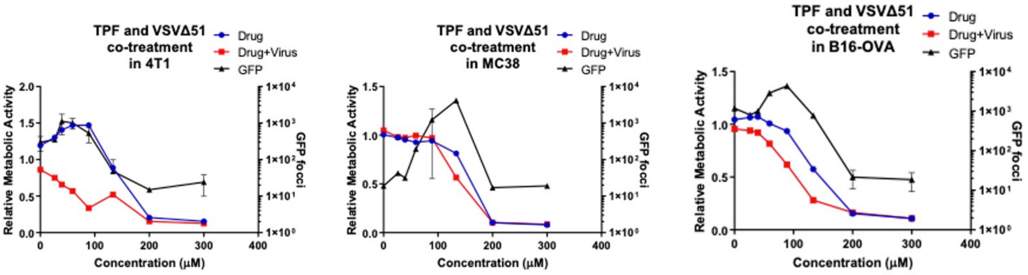

Figure S2: Tepilamide Fumarate promotes VSVΔ51 infection in several murine cancer cells.

A panel of murine cell lines were treated with TPF with a range of concentrations then infected with VSVΔ51 expressing GFP (MOI 0.05). Corresponding fluorescent images were taken 24-48 hours post infection as shown in (A). GFP was quantified with a Cellomics ArrayScan® VTI HCS Reader (Thermo Fisher Scientific). Cell viability was assessed in all cells tested 48 hours after infection. The results were adjusted relative to the average values recorded for the respective uninfected and untreated cells as shown in (B).

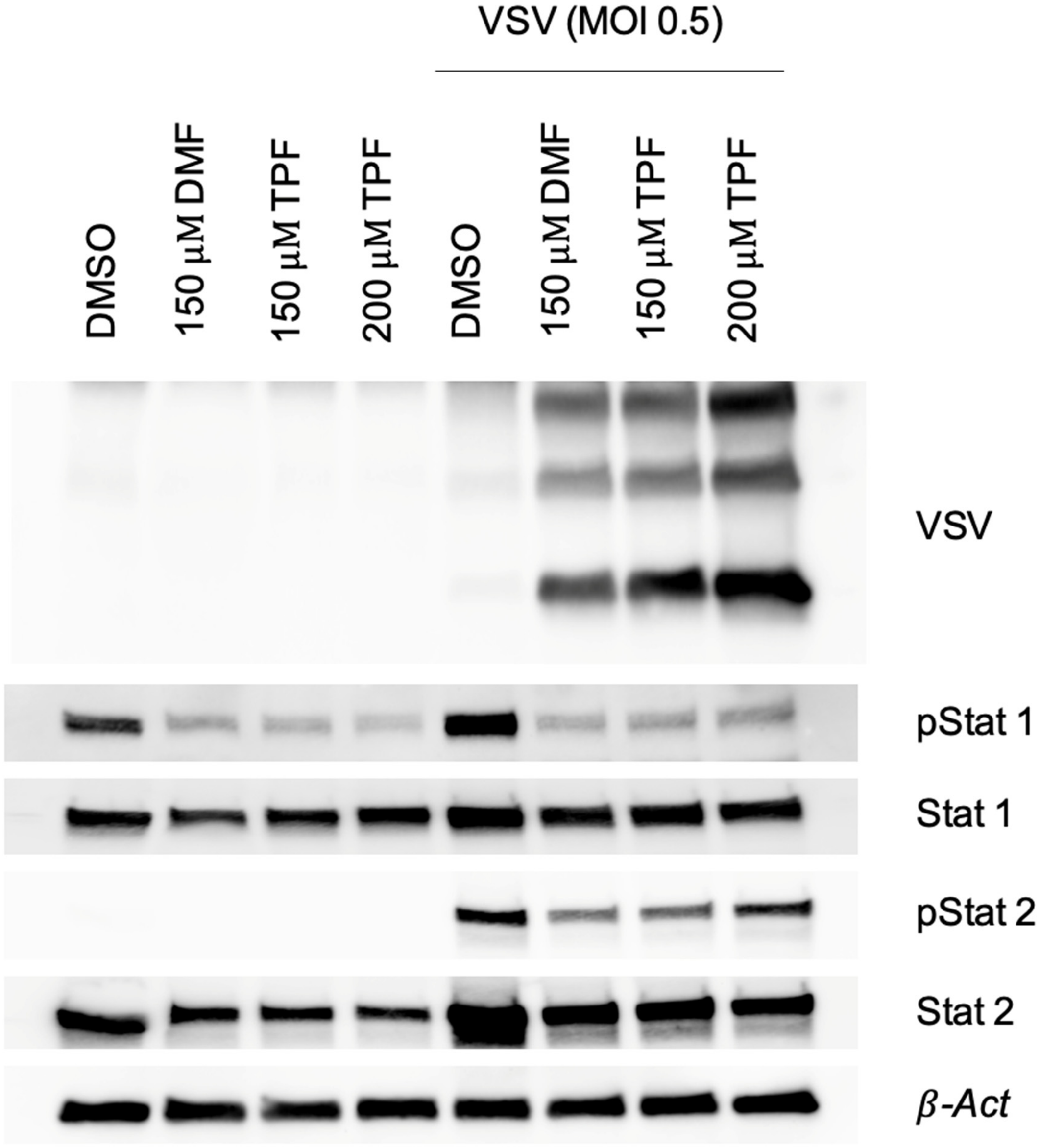

Figure S3: TPF decrease activation of STAT1 and STAT2.

STAT1 and STAT2 phosphorylation status was analyzed by western blot using whole cell lysates from 786-0 cells treated with TPF or DMF with indicated concentration then infected with VSVΔ51 for 24 hours.

Table S1: Clinical characteristics of patients-derived tumor specimens used in the study.

Specimen	ID	Age	Biological Sex	Pathology Report
Lung	GTC 565	61	Female	Invasive mucinous adenocarcinoma
Lung	GTC 564	73	Male	Squamous cell carcinoma
Pancreas	GTC 569	65	Female	Ductal adenocarcinoma
Ovarian	GTC 567	51	Female	High grade serous carcinoma
Pancreas	GTC 572	82	Female	Well differentiated neuroendocrine tumour
Colon	GTC 586	76	Male	Moderately differentiated invasive colonic adenocarcinoma

Table S2: List of cell lines used in this study.

Cell lines	Organism	Tissue	Cell type	Growth media	Vendor	Catalog number
Vero	African green monkey	Kidney		DMEM	ATCC	CCL-81
786-0	Human	Kidney	Renal cell adenocarcinoma	DMEM	ATCC	CRL-1932
CT26.wt	Mouse	Colon	Carcinoma	DMEM	ATCC	CRL-2638
JIMT-1	Human	Breast	Carcinoma	DMEM	DSMZ	ACC-589
HT1080	Human		Fibrosarcoma	DMEM	ATCC	CCL-121
A549	Human	Lung	Carcinoma	RPMI	ATCC	CCL-185
HepG2	Human	Liver	Carcinoma	DMEM	ATCC	HB-8065
MRC5	Human	Lung	Fibroblast	DMEM	ATCC	CCL-171
Jurkat	Human	Blood	Lymphocyte	RPMI		
B16-OVA	Mouse	Skin	Melanoma	RPMI	**	

** Provided by Dr. Yonghong Wan (McMaster University, Hamilton, Ontario, Canada)

Table S3: List of primers used in this study.

Model	Gene	Forward primer (5' to 3')	Reverse primer (5' to 3')
VSV	M	ATACTCAGATGTGGCAGCCG	GATCTGCCAATACCGCTGGA
	N	GATAGTACCGGAGGATTGACG	TCAAACCATCCGAGCCATTC
Human	GAPDH	ACAGTCAGCCGCATCTTCTT	GTTAAAAGCAGCCCTGGTGA
	IFN-β	CATTACCTGAAGGCCAAGGA	CAGCATCTGCTGGTTGAAGA
	MX2	GAACGTGCAGCGAGCTTGTC	AAGGCTTGTGGGCCTTAGAC
	CCL5	GCAGTCGTCCACAGGTCAAG	TCTTCTCTGGGTTGGCACAC
	IL-6	ACCCCCAATAAATATAGGACTGGA	GAAGGCGCTTGTGGAGAAGG
	IFITM1	CCGTGAAGTCTAGGGACAGG	GGTAGACTGTCACAGAGCCG
